# SOD2 deregulation enhances migration, invasion and has poor prognosis in salivary adenoid cystic carcinoma

**DOI:** 10.1038/srep25918

**Published:** 2016-05-16

**Authors:** Boyang Chang, Hang Yang, Yuan Jiao, Kefeng Wang, Zhonghua Liu, Peihong Wu, Su Li, Anxun Wang

**Affiliations:** 1 Sun Yat-sen University Cancer Center; State Key Laboratory of Oncology in South China; Collaborative Innovation Center for Cancer Medicine, Guangzhou, Guangdong 510060, P. R. China; 2 Department of Oral and Maxillofacial Surgery, The First Affiliated Hospital, Sun Yat-Sen University, Guangzhou, Guangdong 510080, P. R. China; 3 Clinical Trial Center, Sun Yat-sen University Cancer Center, Guangzhou, Guangdong 510060, P. R. China; 4 Department of Medical Oncology, Sun Yat-sen University Cancer Center, Guangzhou, Guangdong 510060, P. R. China; 5 Department of Image-guided Minimally Invasive Therapy, Sun Yat-sen University Cancer Center, Guangzhou, Guangdong 510060, P. R. China

## Abstract

This study aimed to investigate the role of SOD2 in the progression and metastasis of salivary adenoid cystic carcinoma (SACC). We analyzed the expression of SOD2 in 50 SACC patients. Then, the effects and mechanism of SOD2 on cell metastasis in a pair of different metastatic potential cell lines was investigated. SOD2 was deregulated in patients with SACC. Up-regulation of SOD2 was associated with distant metastasis and reduced overall survival and disease free - survival. Compared to SACC-83 cells (lower metastasis ability), SACC-LM cells (higher metastasis ability) had higher SOD2 activity and intracellular H_2_O_2_ concentrations, and protein levels of pERK1/2 and Slug, but had similar catalase protein level and activity. In SACC-LM, reducing the expression of SOD2 by SiRNA inhibited the metastasis ability and reduced the SOD2 activities, intracellular H_2_O_2_ concentrations, and protein levels of pERK1/2 and Slug. These effects were revised in SACC-83 after SOD2 overexpression. Moreover, in SACC-83, treated with H_2_O_2_, the metastasis was enhanced accompanied by increased protein levels of pERK1/2 and Slug. We confirmed that SOD2 play an important role in the development and prognosis of SACC and SOD2-dependent production of H_2_O_2_ contributes to metastasis of SACC through the ERK-Slug signaling pathway.

Salivary adenoid cystic carcinoma (SACC) is a malignant tumor that most often arises from the secretory epithelial cells of salivary glands. It accounts for 25% of malignant tumors in the major salivary glands^1^. The biological properties of this carcinoma include aggressive growth, perineural invasion, distant metastases and high rates of local recurrence. Many studies have focused on identifying molecules associated with SACC progression and metastasis^2^,^3^. In our previous studies, we also found that MicroRNA-181a and Bmi-1 contributed an important role in the development of SACC.

Manganese superoxide dismutase (MnSOD, or SOD2) in the mitochondria efficiently converts superoxide to hydrogen peroxide, which may be broken down further into water and dioxygen by other enzymatic (catalase) and non-enzymatic antioxidants^4^. Of the three major forms of SOD, SOD2 is the most effective antioxidant enzyme in mitochondria and is important in protecting cells from reactive oxygen species (ROS)–induced oxidative damage^5^. Emerging evidence has implicated ROS and the activation of redox-sensitive signaling pathways in invasion and migration^6^. Till now, many researches had found that SOD2 was deregulated in the development of many cancer types^7^,^8^,^9^,^10^. SOD2 overexpression was related to metastatic phenotype in cancers^7^,^11^,^12^. The SOD2-dependent production of H_2_O_2_ increases expression of matrix metalloproteinase (MMP) family members, and this increase strongly related to enhanced metastasis^13^,^14^. These observations led us to hypothesize that SOD2 deregulation may be related to the development and metastasis of SACC.

To investigate the role of SOD2 in the development of SACC, we detected the expression of SOD2 in SACC with immunohistochemistry. Then, the effects and mechanism of SOD2 on cell migration and invasion in a pair of different metastatic potential cell lines (SACC-LM and SACC-83) was investigated. Our findings suggest that SOD2 play an important role in the development and prognosis of SACC and that SOD2 may enhance the migration and invasion of SACC through the H_2_O_2_-dependent ERK-Slug pathway.

## Results

### SOD2 deregulation in the development of SACC

**Figure 1:**
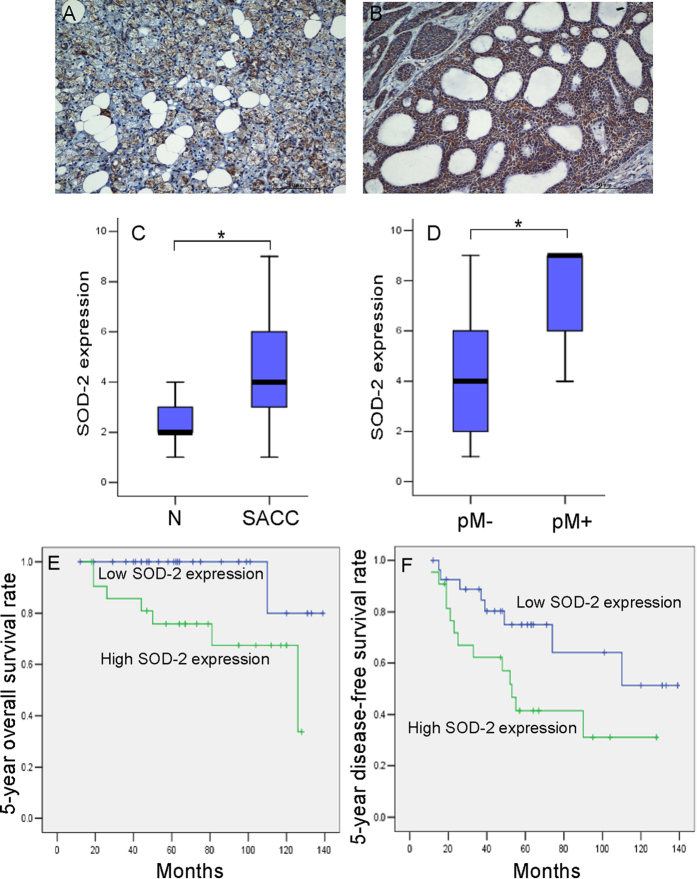
The deregulation of SOD2 in the development of SACC tissue. Immunohistochemical analyses for SOD2 expression were performed in normal salivary gland (**A**) and SACC samples (**B**). (Scale bar: 50 μm) Box plots compare the expression of SOD2 between 20 normal salivary glands and 50 SACC samples (**C**), *P* = 0.000), and SACC with (n = 8) or without (n = 42) distant metastasis (**D**), *P* = 0.000). Boxes represent the 25^th^ and 75^th^ percentiles. The lines in the middle of the box represent medians. *P < 0.001. Patients were divided into SOD2 low expression group (n = 28) and SOD2 high expression group (n = 22). The 5-year overall survival rate (**E**), *P* = 0.017) and 5-year disease-free survival rate (**F**), *P* = 0.038) were significantly higher in SOD2 low expression group. *P < 0.05. Student’s T test was used to compare the difference between groups (**C**,**D**). Survival curves were plotted using the Kaplan-Meier method and compared with the log-rank test (**E**,**F**).

To confirm the relationship between SOD2 deregulation and the development of SACC, immunohistochemistry analyses for SOD2 were performed on tissues samples from 50 SACC patients and from 20 patients with normal salivary glands. SOD2 protein was located in the cytoplasm and the expression of SOD2 was significantly higher in primary cancer tissues than in normal salivary gland tissues (Fig. 1A–C, *P* < 0.001). Among SACC cases, SOD2 expressions were significantly higher in SACC samples with positive distant metastasis status (pM+) than in those with negative status (pM−) (Fig. 1D, *P* < 0.001). Then, we analyzed the association between SOD2 expression and clinicopathologic characteristics in SACC patients and found that higher SOD2 expression were associated with vital status (*P* = 0.02) as well as with the presence of distant metastasis (*P* = 0.02) in SACC patients (Table 1). SOD2 expression was not associated with age, sex, recurrence, tumor stage, clinical stage, or tumor site.

### The prognostic value of SOD2 deregulation in SACC patients

To clarify the prognostic value of SOD2 expression in SACC patients, we examined the relationship between SOD2 expression and long-term outcomes. As illustrated in Fig. 1E, a striking difference in 5-year overall survival (OS) was observed between the high SOD2 expression group (mean survival = 100.2 months) and the low SOD2 expression group (mean survival = 133.2 months, *P* = 0.017). A statistically significant difference in survival was also observed between 5-year disease free survival (DFS) and SOD2 expression (Fig. 1F, *P* = 0.038).

To further evaluate the association between SOD2 expression and clinicopathological factors on the prognosis of SACC patients, univariate and multivariate analyses were carried out. For 5-year overall survival, both univariate and multivariate analysis indicated that only SOD2 expression significantly predicted survival for patients with SACC. However, for 5-year disease-free survival, both univariate and multivariate analyses indicated that SOD2 expression, distant metastasis, and recurrence were independent predictors (Table 2). Thus, our findings indicate that SOD2 expression is significantly associated with the prognosis of SACC.

### The expressions of SOD2 in pair SACC cell lines with different migration and invasion ability

**Figure 2:**
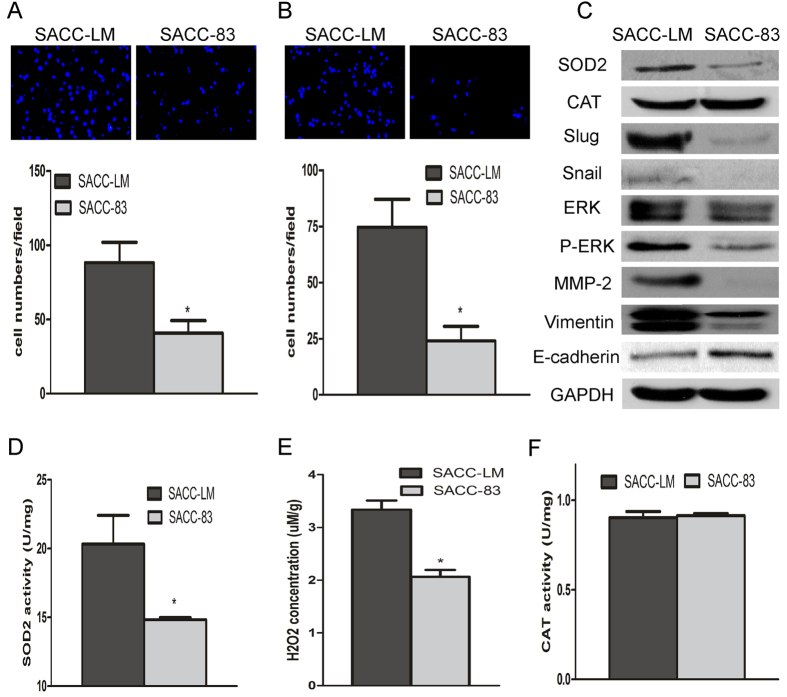
The expression of SOD2 in pair SACC cell lines with different migration and invasion ability. Migration and invasion of SACC cells were assessed using a transwell migration and invasion assay. Migration (**A**) and invasion (**B**) were significantly higher in SACC-LM cells than in SACC-83 cells. SOD2 protein expression and activity were assessed with Western blot (**C**) and SOD activity assays (**D**), respectively. SOD2 expression and activity were significantly higher in SACC-LM cells than in SACC-83 cells. H_2_O_2_ concentrations were measured as described in the text. SACC-LM cells produced significantly more H_2_O_2_ than SACC-83 cells (**E**). Concentrations of CAT (catalase) and activity, assessed with Western blot (**C**) and a CAT activity assay (**F**), respectively, did not differ between the two cell lines (**C**,**F**). Vimentin, Slug, Snail, ERK, p-ERK, and MMP-2 protein expression were higher in SACC-LM cells, and E-cadherin protein expressions were lower in SACC-83 cells (**C**). *P < 0.05.

As shown in Fig. 2A,B, the migration and invasion abilities of SACC-LM were significantly higher than those of SACC-83, as detected by transwell invasion assays. The protein levels and activity of SOD2 in SACC-LM cells were significantly higher than those in SACC-83 cells (Figs 2C,D and S2). Intracellular H_2_O_2_ production was 1.6-fold higher in SACC-LM cells than in SACC-83 cells (Fig. 2E). However, catalase protein levels and activity did not differ between the SACC-LM and SACC-83 cell lines (Fig. 2F). We also found that SACC-LM cells expressed higher level of metastatic related gene, such as Vimentin, Slug, Snail, p-ERK, and MMP-2, and lower in E-cadherin than those in SACC-83 cells (Fig. 2C). These results implicate elevated SOD2 expression, concomitant with an increase in H_2_O_2_ production, in SACC cells with higher migration and invasion ability.

### SOD2 knockdown inhibits migration and invasion of SACC

**Figure 3:**
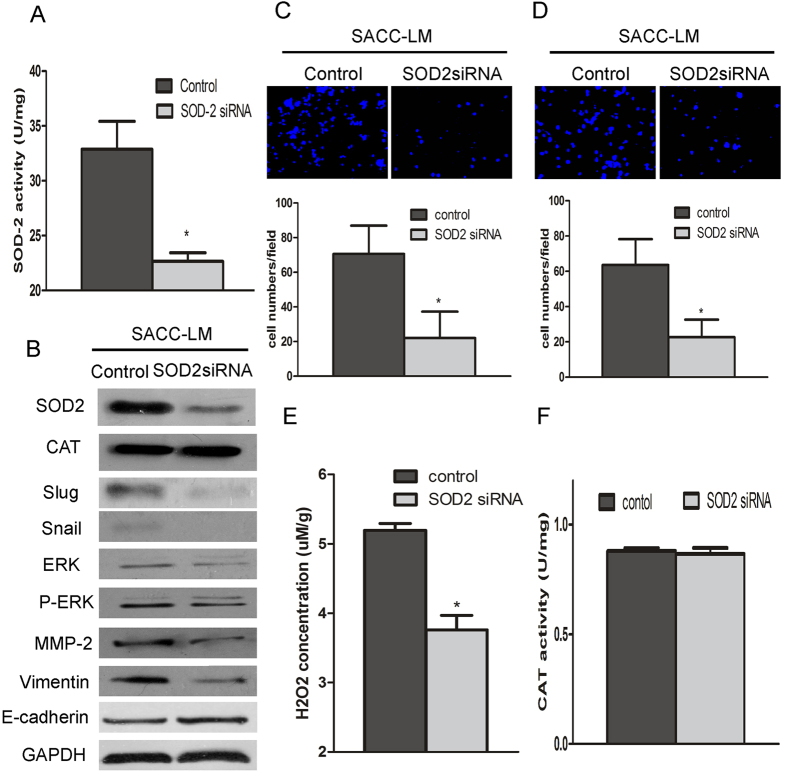
SOD2 knockdown inhibits the migration and invasion of SACC cells. To characterize the function of SOD2 in aiding metastasis, SOD2 siRNA was transfected into SACC-LM cells using Lipofectamine^TM^ RNAiMAX. Cells were collected and tested 24 h after transfection. SOD2 protein concentrations and activities were significantly lower in the SOD2 siRNA-transfected SACC-LM cells than in the control transfected cells (**A**,**B**). SOD2 knockdown inhibited the migration and invasion of SACC-LM cells (**C**,**D**). H_2_O_2_ production was significantly lower in SACC-LM cells after transfection with the SOD2 siRNA (**E**). CAT protein concentrations and activity in SACC-LM cells did not differ after transfection with SOD2 siRNA (**B**,**F**). Vimentin, Slug, Snail, ERK, p-ERK, and MMP-2 protein concentrations were lower in SACC-LM cells but E-cadherin protein concentrations were higher with SOD2 knockdown (**B**). *P < 0.05.

To describe the function of SOD2 in promoting metastasis, we knocked down the expression of SOD2 by siRNA interference. As shown in Fig. 3A,B, both the protein level and activity of SOD2 were obviously decreased in SACC-LM cells after transfection with SOD2 siRNA. The SACC-LM cells transfected with SOD2 siRNA had significantly decreased migration and invasion ability than those of the cells transfected with the control vector (Fig. 3C,D). Furthermore, SOD2 knockdown reduced H_2_O_2_ production in SACC-LM cells (Fig. 3E). No difference of the catalase was found between cell lines transfected with control siRNA and SOD2 siRNA (Fig. 3F). Moreover, after knockdown SOD2, the expression of metastatic related gene, such as Vimentin, Slug, Snail, p-ERK, and MMP-2, were obviously inhibited and E-cadherin was higher in SACC-LM cell transfected with SOD2 siRNA than transfected with control siRNA (Fig. 3B). Moreover, we found that SOD2 knockdown resulted in reduced the cell proliferation rate, but no evident effect in apoptosis (Figs S3A and S4A). These results indicated that SOD2 knockdown, concomitant with a reduction in H_2_O_2_ production, decreased the migration and invasion of SACC.

### SOD2 overexpression promotes migration and invasion of SACC

**Figure 4:**
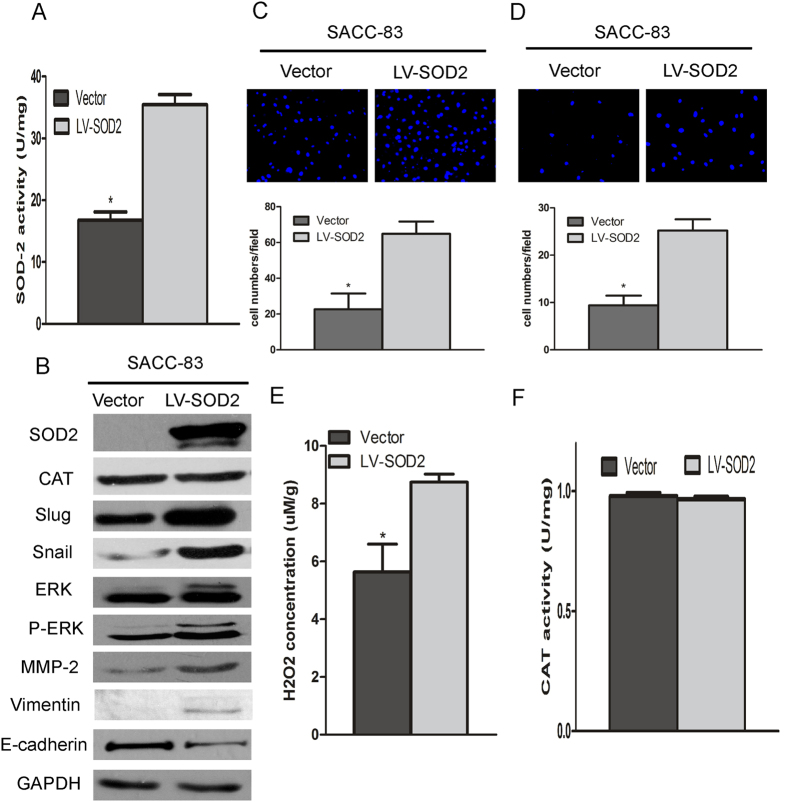
SOD2 overexpression promotes the migration and invasion SACC. To characterize the function of SOD2 in aiding metastasis, lentivirus containing SOD2 overexpression was transfected into SACC-83 cells. SOD2 protein concentrations and activities were significantly higher in the SOD2 overexpression-transfected SACC-83 cells than in the vector control transfected cells (**A**,**B**). SOD2 overexpression promoted the migration and invasion of SACC-83 cells (**C**,**D**). SACC-83 cells significant increased production of H_2_O_2_ after SOD2 overexpression (**E**). CAT protein concentrations and activity in SACC-83 cells did not differ after SOD2 overexpression (**B**,**F**). Vimentin, Slug, Snail, ERK, p-ERK, and MMP-2 protein concentrations were higher in SACC-83 cells, and E-cadherin protein concentrations were lower with SOD2 overexpression (**B**). *P < 0.05.

To further investigate the role of SOD2 in promoting metastasis, SACC-83 cells were transfected with the lentiviral construct containing SOD2. As shown in Fig. 4A,B, the protein expression and activity of SOD2 were increased. SACC-83 cells transfected with SOD2 lentiviral had significantly increased migration and invasion than those of cells transfected with the control vector (Fig. 4C,D). In addition, SOD2 overexpression in SACC-83 cells increased H_2_O_2_ production (Fig. 4E), but no effect on the catalase expression and activity (Fig. 4F). Futhermore, after overexpression of the SOD2 in SACC-83 cells, the metastatic related gene, such as Vimentin, Slug, Snail, p-ERK and MMP-2 were obviously higher, and E-cadherin protein was lower (Fig. 4B). Moveover, we found that there is significantly higher proliferation capacity, but no difference in apoptosis after SOD2 overexpression in SACC-83 cells (Figs S3B and S4B). ERK 1/2 inhibitor can block SOD2-induced metastasis and decrease its related protein expression (e.g., Slug, Snail, Vimentin) (Fig. S5).These results indicated that SOD2 overexpression increased production of H_2_O_2_, leading to increases in the migration and invasion of SACC.

### SOD2-Dependent H_2_O_2_ Production Induced Migration and Invasion of SACC

**Figure 5:**
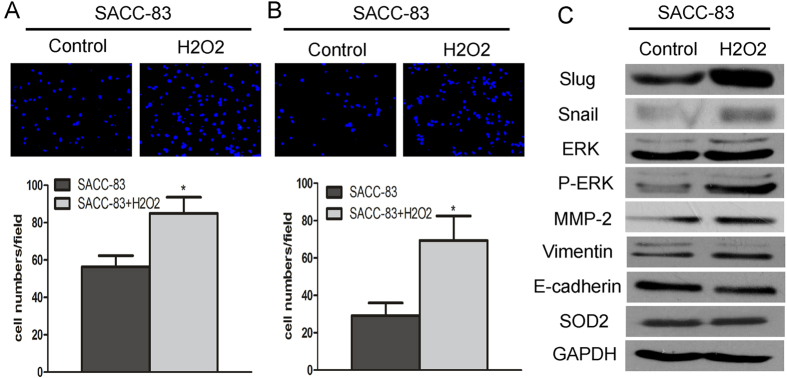
SOD2-dependent production of H_2_O_2_ increases migration and invasion of SACC. To confirm that H_2_O_2_ production induces migration and invasion of SACC, SACC-83 cells were treated with H_2_O_2_ for 24 h. The migration and invasion of SACC-83 cells were significantly higher after treatment with 25 μM H_2_O_2_ once every 6 h (**A**,**B**). Adding H_2_O_2_ increased the protein concentrations of Vimentin, Slug, Snail, ERK, p-ERK, and MMP-2 and decreased E-cadherin protein concentrations and had no affection on expression of SOD2 (**C**).

To further confirm that SOD2-dependent H_2_O_2_ production induces migration and invasion of SACC, the SACC-83 cells, which have lower SOD2 activity and H_2_O_2_ production than SACC-LM cells, were treated with 25 μM H_2_O_2_ once every 6 h. Significantly increased migration and invasion were detected in treated SACC-83 (Fig. 5A,B). Moreover, H_2_O_2_ treatment in SACC-83 led to a corresponding increase in metastatic related gene, such as Vimentin, Slug, Snail, p-ERK, MMP-2, and a decrease in E-cadherin concentrations, while no effect on SOD2 expression was found in SACC-83 cells after treated with H_2_O_2_ (Fig. 5C). ERK 1/2 inhibitor can block H_2_O_2_ -induced metastasis (Fig. S6). To gain more insight into the function of H_2_O_2_, SACC-LM cells were treated with catalase, which can remove H_2_O_2_ into H_2_O and O_2_. Migration and invasion of SACC-LM were significantly inhibited after treatment with 600 U catalase (Fig. S7). These results suggest that H_2_O_2_ production may be needed for the migration and invasion effects induced by SOD2 in SACC.

## Discussion

Many cancers show increases in SOD2 expression during progression to metastatic disease^15^,^16^,^17^. Our previously studies found that SOD2 expression is consistently elevated in tongue cancer specimens and that SOD2 expression is significantly higher in lymph node metastases than in paired primary tumors^7^,^11^,^17^. In the present research, high expression of SOD2 was found in SACC and associated with distant metastasis in SACC. Further, multivariate Cox regression analysis revealed that high SOD2 expression predicted poor overall survival and decreased disease-free survival. Elevated SOD2 expression is also correlated with increased metastasis *in vitro* studies^11^,^14^. SACC-LM cell line with higher metastatic and invasive ability displayed apparently higher SOD2 protein and activity levels than SACC-83 cells with lower metastatic and invasive ability. Migration and invasion were significantly increased after overexpression SOD2 in SACC-83 and significantly inhibited in SACC-LM cells on SOD2 knockdown. These findings suggested that SOD2 play an important role in the development and prognosis of SACC and that increased SOD2 protein expression contributed to the invasive and metastatic capacity of SACC.

Several researchers have hypothesized that SOD2 overexpression promotes metastasis by increasing the steady-state concentration of H_2_O_2_^13^,^18^,^19^. In the present study, we also found that the higher the concentration of SOD2 and activity, the higher the intracellular H_2_O_2_ concentration and the higher the cellular migration and invasion. The intracellular H_2_O_2_ concentration fluctuated along with the SOD2 expression levels. Furthermore, the protein concentration and activity of catalase that can remove H_2_O_2_ were stable between SACC-LM and SACC-83 cells, also in SACC cells overexpression or knockdown of SOD2. Treatment with H_2_O_2_ obviously enhanced the migration and invasion ability of SACC-83 cells with lower H_2_O_2_, but migration and invasion ability were significantly reduced in SACC-LM cells with higher H_2_O_2_ after treatment with catalase. These results indicate that SOD2-mediated invasion and metastasis of SACC cells may depend on H_2_O_2_ concentrations and that the intracellular accumulation of H_2_O_2_ must be related to high SOD2 expression but not to catalase activity.

Epithelial-mesenchymal transition (EMT), in which epithelial cells acquire mesenchymal-like properties, is thought to be a critical step in the induction of tumor metastasis^20^,^21^. Snail is one of the master regulators that promotes EMT and mediates invasiveness as well as metastasis in many different types of malignant tumors. Loss of E-cadherin and gain of Vimentin are hallmark of the invasive phase of cancer^22^,^23^. In our study, overexpression of SOD2 increases the motility and invasive properties of SACC-83 cells, which is concurrent with the increased expression of mesenchymal markers (Vimentin) and Snail, the decreased expression of epithelial markers (E-cadherin). The opposite results were obtained with knockdown of SOD2 in SACC-LM cells. Taken together, our findings demonstrated that SOD2 regulation of SACC migration and invasion may be involved the EMT process.

In our previous study, we found that the ERK target Slug, which is related to the metastasis of SACC cells. Moreover, siRNA-mediated p-ERK knockdown reduced the Snai protein level in SACC cells^2^. Others have also hypothesized that increases in SOD2 concentrations activate ERK and the consequent downstream transcriptional elevations in matrix metalloproteinases, which may be important in tumor progression^13^,^24^,^25^. Here, we found that the protein concentrations in the ERK-Slug pathway (including pERK, Slug) were more highly expressed in SACC-LM cells than in SACC-83 cells and overexpression of SOD2 in SACC-83 increased the expression of these proteins. The opposite reactions were obtained with knockdown of SOD2 in SACC-LM, the expression of these proteins were also significantly increased in SACC-83 after treated with H_2_O_2_. These results indicate that SOD2-dependent intracellular H_2_O_2_ production may activate ERK- Slug signaling and increase the invasive and metastatic capacity of SACC.

From above, we confirmed that SOD2 is important in the development and prognosis of SACC; that SOD2 deregulation is related to migration and invasion in SACC; and that the SOD2-dependent production of intracellular H_2_O_2_ promotes the migration and invasion of SACC, which involves the ERK-Slug signaling pathway. Further investigation of SOD2 involvement in the progression and metastasis of SACC may help identify new therapeutic strategies and improve the survival of patients with this disease.

## Materials and Methods

### Ethic statement

For the use of all clinical materials for research purposes, prior written informed consents from all patients and approval from the Institute Research Ethics Committee of Sun Yat-sen University Cancer Center were obtained (B2013-045-01). All the methods were carried out in accordance with the approved guidelines.

### Patients and samples

In this retrospective study, we collected tissue samples from 50 patients with SACC who had undergone radical resection without preoperative chemotherapy or radiotherapy (Supplementary Table S1) and 20 normal salivary glands from patients undergoing surgery at the Sun Yat-sen University Cancer Center between 1998 and 2010(Supplementary Table S2). Tumors were staged according to Union for International Cancer Control (UICC) system. Survival was calculated from the date of diagnosis to the date of the latest follow-up visit or death. Median duration of follow-up for the 50 patients was 64 months (range, 12 to 139 months).

### Immunohistochemical staining

Immunohistochemistry was performed on 5 mm sections of formalin-fixed, paraffin-embedded tissue samples as previously described^26^. Briefly, the paraffin section was deparaffinized with xylene and rehydrated in alcohol. Antigen retrieval was accomplished with boiling citrate buffer and endogenous peroxidase activity was blocked with 3% H_2_O_2_ followed by staining with anti-SOD2 antibody (1:1000; Atlas, Stockholm, Sweden) overnight at 4 °C. After washing, the sections were incubated with the MaxVision ^TM^ HRP-Polymer anti-Rabbit IHC Kit at room temperature (Maixin, Fuzhou, China), developed with the DAB Horseradish Peroxidase Color Development Kit (Maixin, Fuzhou, China), and counterstained with hematoxylin. Rabbit (DA1E) mAb IgG XP (Cell Signaling Technology, Beverly, MA, USA) was used as an isotype control (Fig. S1).

The degree of immunostaining was scored independently by two observers as the proportion of positively stained tumor cells and by the intensity of staining. The proportion of tumor cells was scored as: 0 (no positive tumor cells), 1 (less than <30% positive tumor cells), 2 (30% to 60% positive tumor cells), and 3 (greater than >60% positive tumor cells). The intensity of staining was graded as: 0 (for no staining), 1 (weak staining, light yellow), 2 (moderate staining, yellow brown) and 3 (strong staining, brown). The staining index was calculated as the staining intensity score times the proportion of positive tumor cells, which produced scores of 0, 1, 2, 3, 4, 6, and 9. Staining index scores of 6 or 9 defined high SOD2 expression, and scores of 4 or less defined low expression of SOD2.

### Cell culture and transfection

The paired cell lines (SACC-LM/SACC-83) were kindly provided by Dr. Shenglin Li. The SACC-LM cell line is more aggressive than the SACC-83 line, in terms of the rate of lung-metastasis^27^,^28^. The SACC-83 and SACC-LM are authentic adenoid cystic carcinoma cell lines^28^. Cells were maintained in RPMI-1640 supplemented with 10% FBS, 100 U/ mL penicillin, and 100 μg/ mL streptomycin at 37 °C in a humidified incubator with 5% CO2. To knockdown the expression of SOD2, SACC-LM cells were transfected with SOD2 siRNA or control siRNA (Genepharma, Shanghai, China) using Lipofectamine^TM^ RNAiMAX transfection reagent (Invitrogen, Carlsbad, CA, USA) according to the manufacturer’s instructions. SOD2 siRNA sequences (sense: GCA UCU GUU GGU GUC CAA GTT). Control siRNA sequences (sense: UUC UCC GAA CGU GUC ACG UTT). To overexpression the expression of SOD2, SACC-83 cell was infected with lentivirus contain SOD2 (NM_000636) and control lentivirus (Genechem, Shanghai, China.

### Western blot analysis

Western blots were performed as described previously, using antibodies specific to extracellular signal- regulated kinase (ERK) 1, Mitogen-activated protein kinase 1/2(pERK1/2), Snail family members (Snai1 and Slug), E-cadherin, Vimentin, Catalase(CAT), MMP-2 (Cell Signaling Technology, Beverly, MA, USA), and SOD2(Atlas, Stockholm, Sweden). GADPH (Sigma-Aldrich, St. Louis, MO, USA) was used as a control.

### *In vitro* cell migration and invasion assays

Transwell assays were performed to assess cell migration and invasion using BD BioCoat Control Cell Culture Inserts or a BD BioCoat BD Matrigel Invasion Chamber, respectively. In brief, cells were seeded in the upper Boyden chambers of the cell culture inserts. After incubating for 24 h (for migration) or 36 h (for invasion), cells remaining in the upper chamber (for migration) or on the upper membrane (for invasion) were carefully removed. Cells adhering to the lower membrane were stained with DAPI in the dark, imaged, and counted using an inverted microscope equipped with a digital camera. Five random fields were captured at 200× magnification under microscope. The number of cells on the bottom surface was compared between groups.

### SOD2 activity

SOD2 activity was measured for its ability to inhibit xanthine/xanthine oxidase-induced cytochrome c reduction in the presence of 5 mmol/L potassium cyanide, which inhibits SOD1 and SOD3 activities^11^. One unit of SOD2 activity was defined as the amount of SOD2 needed to exhibit 50% dismutation of the produced superoxide radical at 25 °C. The final enzyme activity was calculated by normalizing the results to the total protein concentration of the whole protein extract, as determined by the Bio-Rad protein assay (Richmond, CA, USA).

### Catalase activity

The cells were seeded in 6-well plates and harvested for catalase activity analysis after being cultured for 24 h or 48 h. Catalase activity was measured using the Amplex red catalase assay kit (Invitrogen, Carlsbad, CA, USA) following the manufacturer’s instructions.

### Measuring of intracellular H_2_O_2_

The H_2_O_2_ concentration was measured using the Amplex^®^ Red Hydrogen Peroxide/Peroxidase Assay Kit (Invitrogen, Carlsbad, CA, USA), according to the manufacturer’s instructions. The Amplex^®^ Red reagent reacts with H_2_O_2_ in a 1:1 stoichiometry to produce the red-fluorescent oxidation product, resorufin, which can be read on a spectrophotometer at 560 nm.

### Statistical Methods

Data are reported as means ± standard deviations (SD). Differences between groups were assessed with Student’s *t* test. Associations between gene expression and clinical pathologic characteristics were assessed with chi-square tests. Survival curves were plotted using the Kaplan-Meier method and compared with the log-rank test. Cox regression was used for both univariate and multivariate analysis. *P* < 0.05 in all cases was considered statistically significant. All data were analyzed with the Statistical Package for the Social Science (SPSS, Chicago, IL), Version 13.0.

## Additional Information

**How to cite this article**: Chang, B. *et al*. SOD2 deregulation enhances migration, invasion and has poor prognosis in salivary adenoid cystic carcinoma. *Sci. Rep.* **6**, 25918; doi: 10.1038/srep25918 (2016).

## Supplementary Material

Supplementary Information

## Figures and Tables

**Table 1:** Association between SOD2 Expression and Clinicopathologic Characteristics of 50 Patients with Salivary Adenoid Cystic Carcinoma.

	SOD2 expression (n^a^)*P*^b^		
	Low	High	
Sex,			0.41
Male	11	8	
Female	17	14	
Age, (years)			0.08
≤40	19	8	
≪40	9	14	
Tumor stage,			0.12
T_1_ + T_2_	20	11	
T_3_ + T_4_	8	11	
Clinical stage			0.06
I + II	19	9	
III + IV	9	13	
Distant metastasis,			0.02
Negative	27	15	
Positive	1	7	
Recurrence,			0.91
Negative	22	17	
Positive	6	5	
Tumor site,			0.20
Parotid	15	8	
Submandibular	11	12	
Sublingual	4	0	
Vital status,			0.02
Alive	29	13	
Death(tumor-related)	1	7	

^a^number of case.

^b^Chi-square test.

**Table 2:** The association between SOD2 expression and clinicopathological factors on the prognosis of SACC patients by univariate and multivariate analyses.

	Univariate analysis		Multivariate analysis	
	*P*	Regression coefficient (SE)^a^	*P*	Relative risk (95%CI)
Overall survival				
Expression of SOD2	0.047	2.13 (1.07)	0.047	8.39 (1.03 to 68.40)
T stage	0.08	1.48 (0.84)	0.14	…
Clinical stage	0.16	1.19 (0.84)	…	…
Distant metastasis	0.07	1.37 (0.77)	0.38	…
Recurrence	0.84	0.18 (0.84)	…	…
Age	0.21	3.81 (3.06)	…	…
Sex	0.37	−0.75(0.83)	…	…
Disease-free survival				
Expression of SOD2	0.046	0.90 (0.45)	0.046	2.48 (1.02 to 6.03)
T stage	0.06	0.82 (0.44)	0.65	…
Clinical stage	0.12	0.69 (0.44)	…	…
Distant metastasis	<0.001	1.87 (0.47)	<0.001	9.55 (3.50 to 26.04)
Recurrence	<0.001	1.99 (0.47)	<0.001	9.87 (3.73 to 26.11)
Age	0.84	−0.09 (0.45)	…	…
Sex	0.64	−0.22(0.46)	…	…

^a^Factors with *P* values less than 0.1 in the univariate analyses were included in the multivariate model.
