# Supplementary material for: SOD2 deregulation enhances migration, invasion and has poor prognosis in salivary adenoid cystic carcinoma
Source: Sci Rep. 2016 May 16;6:25918. doi: 10.1038/srep25918 (PMC4867643; doi:10.1038/srep25918)
Supplement: Supplementary Information [file srep25918-s1.pdf]

SOD2 deregulation enhances migration, invasion and has poor prognosis in salivary adenoid cystic carcinoma

Boyang Chang[a,b,c,#], Hang Yang[a,d,#], Yuan Jiao[a,b,#], Kefeng Wang [a,d], Zhonghua Liu[c], Peihong Wu[a,e], Su Li[a,b], Anxun Wang[c,*]

[a] Sun Yat-sen University Cancer Center; State Key Laboratory of Oncology in South China; Collaborative Innovation Center for Cancer Medicine, Guangzhou, Guangdong 510060, P. R. China.

[b] Clinical Trial Center, Sun Yat-sen University Cancer Center, Guangzhou, Guangdong 510060, P. R. China.

[c] Department of Oral and Maxillofacial Surgery, The First Affiliated Hospital, Sun Yat-Sen University, Guangzhou, Guangdong 510080, P. R. China.

[d] Department of Medical Oncology, Sun Yat-sen University Cancer Center, Guangzhou, Guangdong 510060, P. R. China.

[e] Department of Image-guided Minimally Invasive Therapy, Sun Yat-sen University Cancer Center, Guangzhou, Guangdong 510060, P. R. China.

# These authors contributed equally to this work.

***Corresponding author**:

Anxun Wang, Department of Oral and Maxillofacial Surgery, The First Affiliated Hospital, Sun Yat-Sen University, 58 Zhongshan road II, Guangzhou 510080, P.R. China; Phone: +86-0-13724896216; E-mail addresses: wang_anxun@aliyun.com

S1 Table. Clinicopathologic characteristics of SACC patients

		No. of cases (%)
**Gender**	Male	19(38%)
	Female	31(62%)
**Age(years)**	≤40	18(36%)
	﹥40	32(64%)
**Tumor stage**	T 1	4(8%)
	T 2	27(54%)
	T 3	6(12%)
	T 4	13(26%)
**Distant metastasis**	Negative	8(16%)
	Positive	42(84%)
**Recurrence**	Negative	39(78%)
	Positive	11(22%)
**Vital status**	Alive	42(84%)
	Death	8(16%)
**Expression of SOD2**	Low expression	28(56%)
	High expression	22(44%)
**Tumor site**	Parotid	23(46%)
	Submandibular	23(46%)
	Sublingual	4(8%)

S2 Table. The histological normal salivary gland tissues used in this study

	**No. of cases**	**Tissues from**
normal sublingual glands	**7**	from sublingual gland cyst
	**1**	from radical neck dissection with tongue squamous cell carcinoma
normal parotid glands	**2**	from radical neck dissection with tongue squamous cell carcinoma
	**2**	from parotid benign hypertrophy
normal submandibular glands	8	from radical neck dissection with tongue squamous cell carcinoma

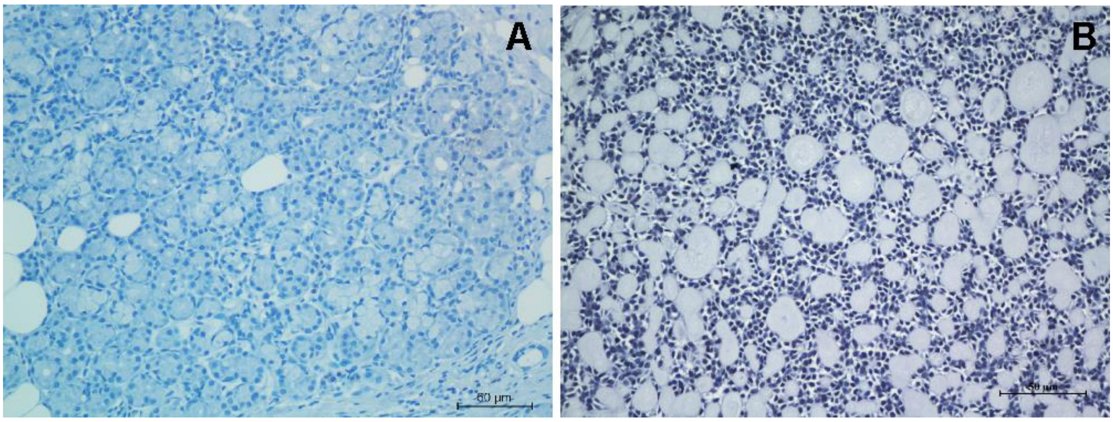

S1 Figure: Isotype control for immunohistochemistry staining of SOD2 expression in SACC and normal salivary gland tissue

Isotype control immunohistochemistry staining for SOD2 expression in A: normal salivary gland; B: salivary gland adenoid cystic carcinoma

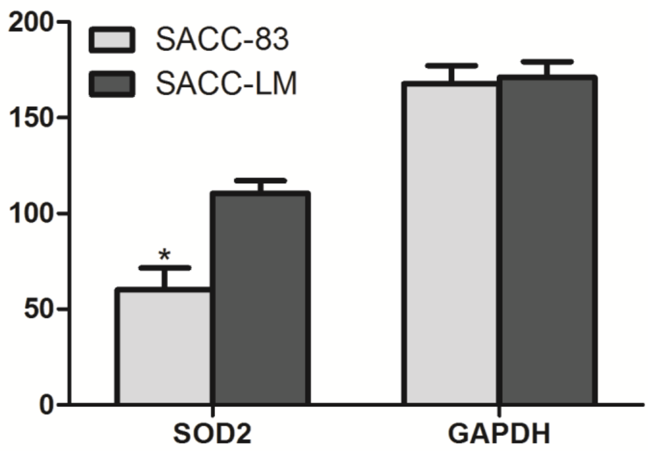

S2 Figure. The quantification of SOD2 expression in SACC cell lines

SOD2 protein expression was quantified by Image J software. SOD2 expression was significantly higher in SACC-LM cells than in SACC-83 cells. *P =0.018

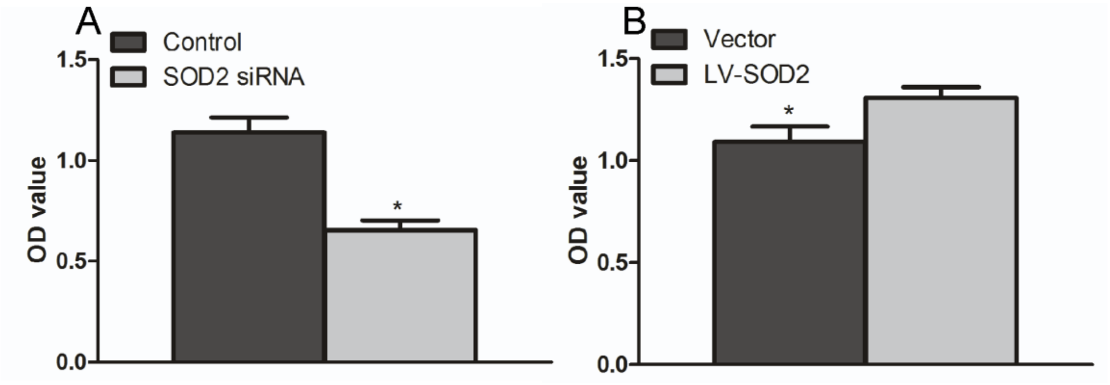

S3 Figure. Cell proliferation of SACC cells was measured using a CCK-8 assay

(A) The cell proliferation rate of SACC-LM line was significantly inhibited after transfection with the SOD2 siRNA. *P = 0.000. (B) The cell proliferation rate of SACC-83 line was significantly enhanced after SOD2 overexpression. *P = 0.007. Student's T test was used to compare the difference between groups.

Cell proliferation was measured by Cell Counting Kit-8 assay according to the manufacturer's instructions. In brief, cells were seeded in 96-well plates at the density of 5 × 10^3 cells per well after incubation for 24 h. Then added 10ul of the CCK-8 solution to each well of the plate and incubate the plate for 2 h in the incubator. The absorbance value of each well was assayed using a plate reader at a wavelength of 450 nm. Experiments were performed in triplicate.

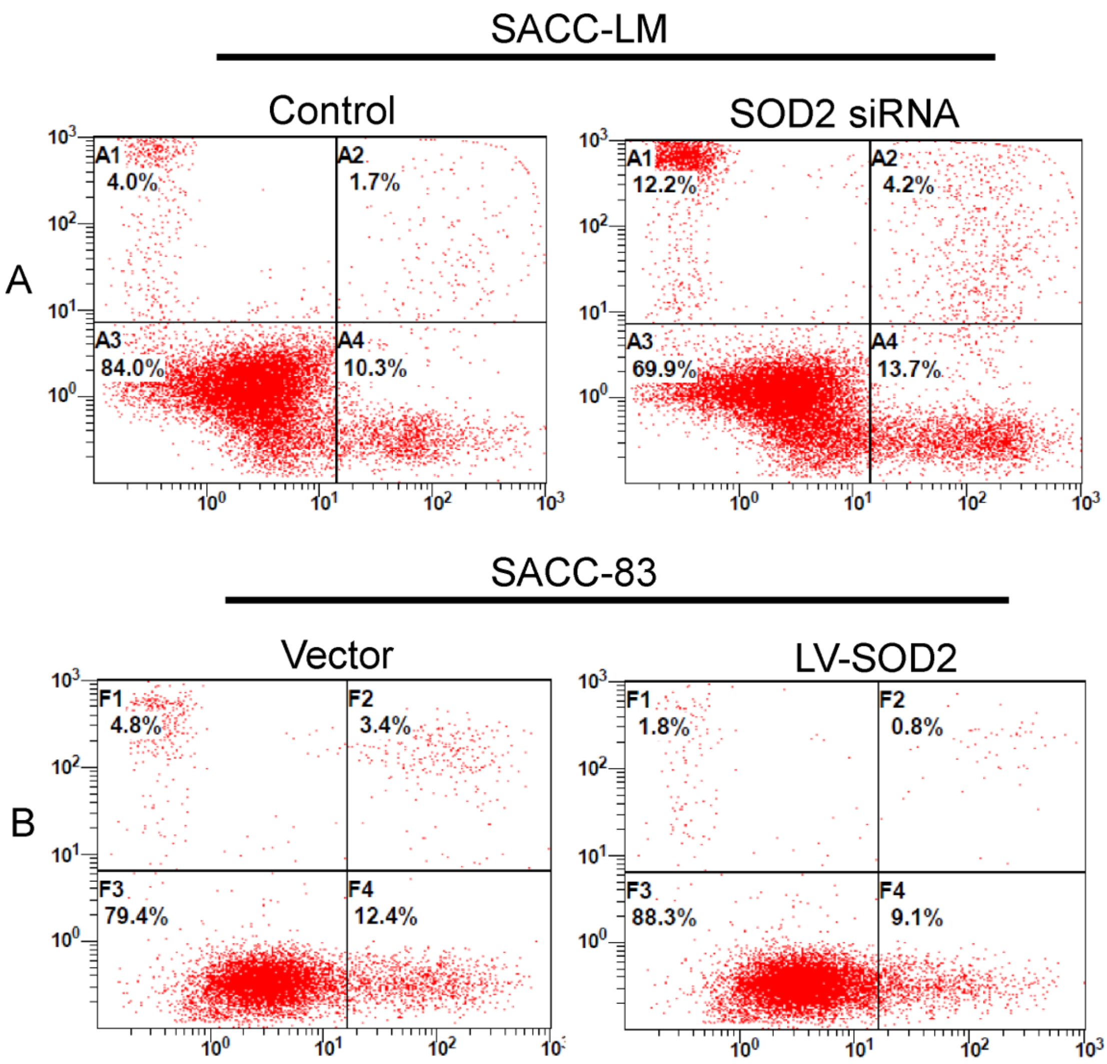

S4 Figure. Apoptosis of SACC cells was detected by Annexin V-FITC-PI flow cytometry assay

(A) The apoptosis rates was 10.3+1.7% in control siRNA compare to 13.7+4.2% in SOD2 siRNA transfected group. (*P*= 0.176). (B) The apoptosis rates was 12.4+3.4% in control lentivirus compare to 9.1+0.8% in SOD2 overexpression group. (*P* = 0.098). Student's T test was used to compare the difference between groups.

Annexin V-FITC-PI flow cytometry assay was used to detect apoptosis. Briefly, the SACC cells were transfection with the SOD2 siRNA for 24h or lentivirus containing SOD2 for 48h. Following collection, the cells were stained with Annexin V-FITC and PI. The apoptotic cells were estimated using a Beckman-Gallios flow cytometer (Beckman-Gallios, Miami, FL, USA). Experiments were performed in triplicate.

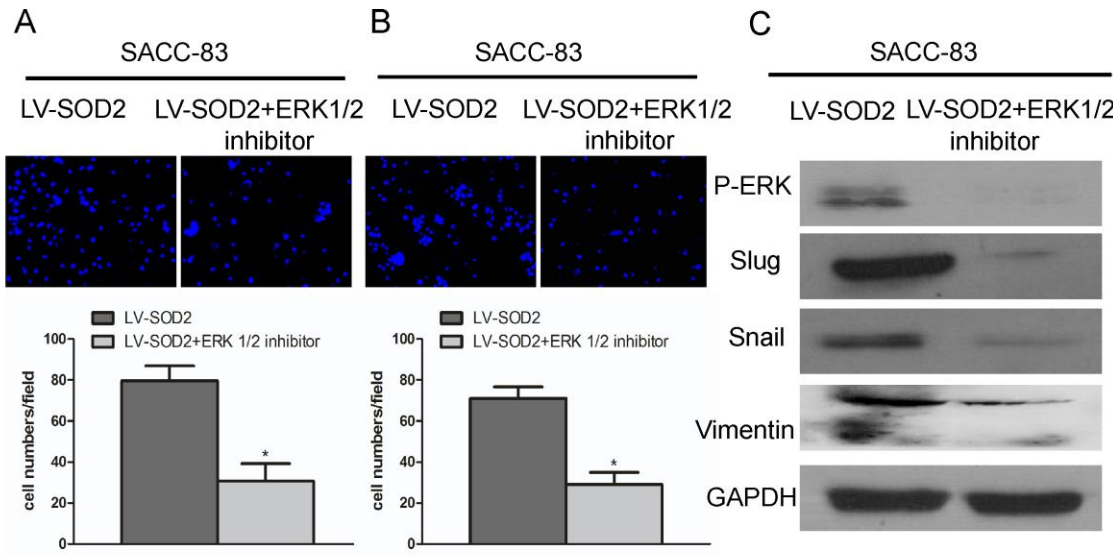

S5 Figure. ERK 1/2 inhibitor blocks SOD2-induced metastasis and decrease its related protein expression in SACC.

ERK 1/2 inhibitor blocked SOD2-induced migration (A, *P* = 0.000) and invasion (B, *P* = 0.000) in SACC-83 cells after overexpression of SOD2. **P* <0.001. (C) Vimentin, Slug, Snail, p-ERK were decreased in SACC-83 cells with SOD2 overexpression after treated with ERK 1/2 inhibitor.

We purchase the ERK 1/2 inhibitor (SCH772984) which was dissolved in DMSO for in vitro studies from the Selleck chemicals. The SACC-83 cells were harvested for following experiments after added SCH772984 for 48h.

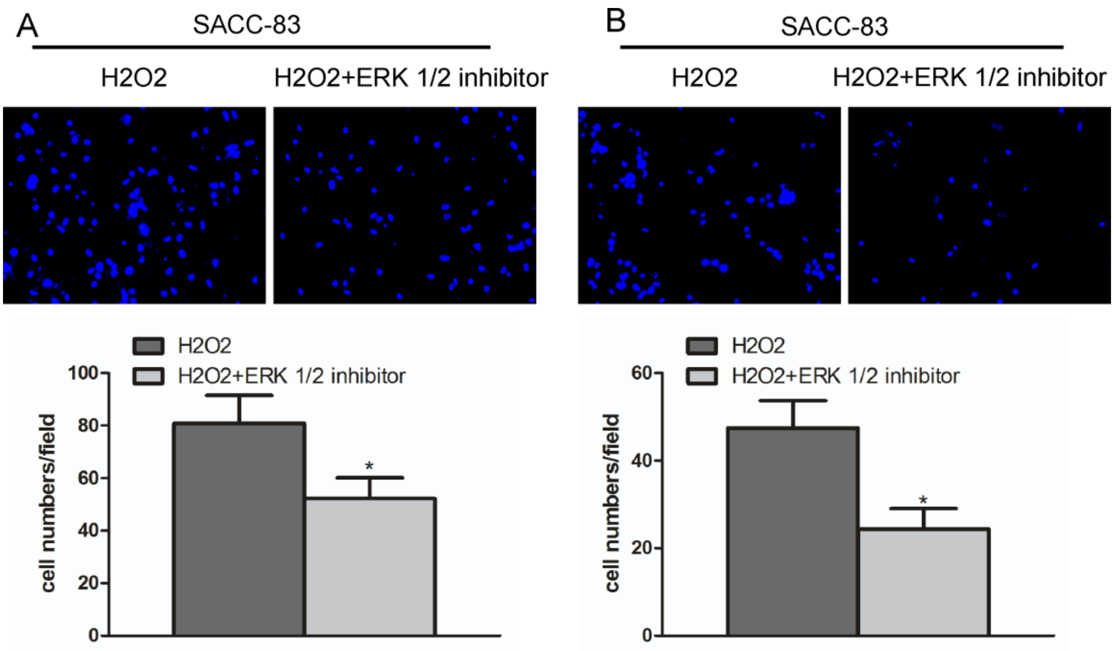

S6 Figure. ERK 1/2 inhibitor blocks H2O2-induced metastasis in SACC.

ERK 1/2 inhibitor blocked H2O2-induced migration (A, *P* = 0.0013) and invasion (B, *P* = 0.002) in SACC-83 cells after treated with H2O2. **P* <0.05.

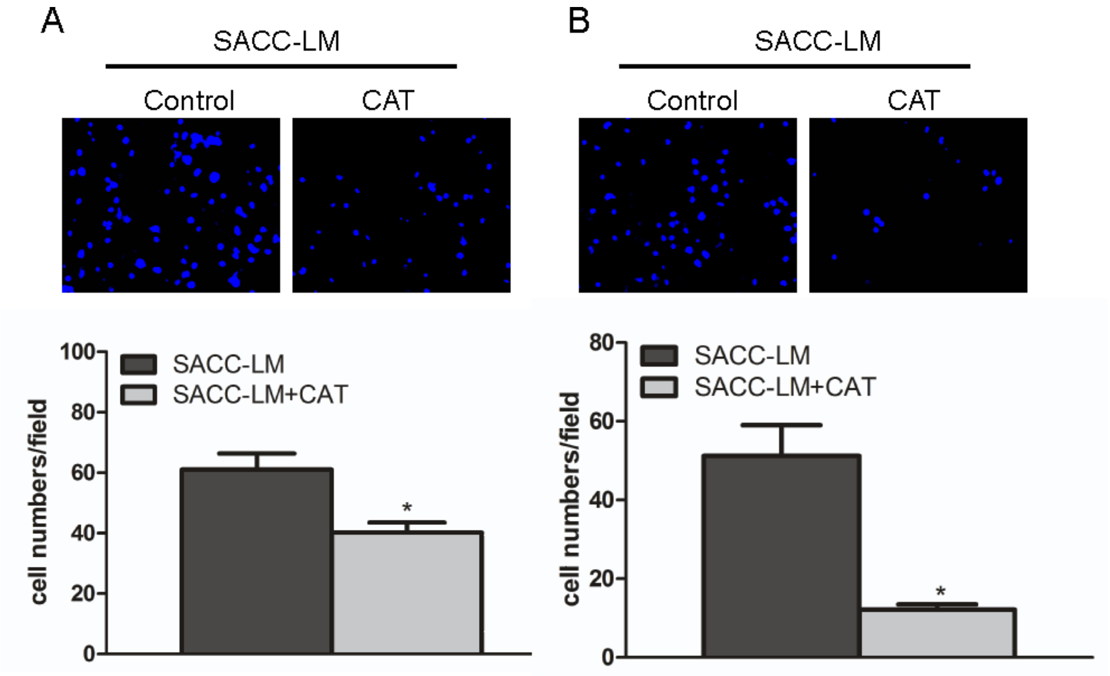

S7 Figure: Catalase inhibits both the migration and invasion of SACC

To gain more insight into the function of H_2O_2, SACC-LM cells were treated with catalase,

which can remove of H_2O_2. The migration (A, *P*=0.000) and invasion (B, *P*=0.000) of SACC-LM were significantly inhibited after treatment with 600 U catalase. **P* <0.001.
